# Distance matters: barriers to antenatal care and safe childbirth in a migrant population on the Thailand-Myanmar border from 2007 to 2015, a pregnancy cohort study

**DOI:** 10.1186/s12884-021-04276-5

**Published:** 2021-12-02

**Authors:** Eric Steinbrook, Myo Chit Min, Ladda Kajeechiwa, Jacher Wiladphaingern, Moo Kho Paw, Mu Paw Jay Pimanpanarak, Woranit Hiranloetthanyakit, Aung Myat Min, Nay Win Tun, Mary Ellen Gilder, François Nosten, Rose McGready, Daniel M. Parker

**Affiliations:** 1 University of Michigan Medical School, University of Michigan, Ann Arbor, MI USA; 2 Shoklo Malaria Research Unit, Mahidol-Oxford Tropical Medicine Research Unit, Faculty of Tropical Medicine, Mahidol University, Mae Sot, Tak Province Thailand; 3 Centre for Tropical Medicine and Global Health, Nuffield Department of Medicine, University of Oxford, Oxford, UK; 4 Population Health and Disease Prevention, University of California-Irvine, Irvine, CA USA; 5 Epidemiology and Biostatistics, University of California-Irvine, Irvine, CA USA

**Keywords:** Pregnancy, Prenatal care, Maternal health, Malaria, Healthcare delivery, Access to care, Geography

## Abstract

**Background:**

Antenatal care and skilled childbirth services are important interventions to improve maternal health and lower the risk of poor pregnancy outcomes and mortality. A growing body of literature has shown that geographic distance to clinics can be a disincentive towards seeking care during pregnancy. On the Thailand-Myanmar border antenatal clinics serving migrant populations have found high rates of loss to follow-up of 17.4%, but decades of civil conflict have made the underlying factors difficult to investigate. Here we perform a comprehensive study examining the geographic, demographic, and health-related factors contributing to loss to follow-up.

**Methods:**

Using patient records we conducted a spatial and epidemiological analysis looking for predictors of loss to follow-up and pregnancy outcomes between 2007 and 2015. We used multivariable negative binomial regressions to assess for associations between distance travelled to the clinic and birth outcomes (loss to follow-up, pregnancy complications, and time of first presentation for antenatal care.)

**Results:**

We found distance travelled to clinic strongly predicts loss to follow-up, miscarriage, malaria infections in pregnancy, and presentation for antenatal care after the first trimester. People lost to follow-up travelled 50% farther than people who had a normal singleton childbirth (a ratio of distances (DR) 1.5; 95% confidence interval (CI): 1.4 – 1.5). People with pregnancies complicated by miscarriage travelled 20% farther than those who did not have miscarriages (DR: 1.2; CI 1.1–1.3), and those with *Plasmodium falciparum* malaria in pregnancy travelled 60% farther than those without *P. falciparum* (DR: 1.6; CI: 1.6 – 1.8). People who delayed antenatal care until the third trimester travelled 50% farther compared to people who attended in the first trimester (DR: 1.5; CI: 1.4 – 1.5).

**Conclusions:**

This analysis provides the first evidence of the complex impact of geography on access to antenatal services and pregnancy outcomes in the rural, remote, and politically complex Thailand-Myanmar border region. These findings can be used to help guide evidence-based interventions to increase uptake of maternal healthcare both in the Thailand-Myanmar region and in other rural, remote, and politically complicated environments.

**Supplementary Information:**

The online version contains supplementary material available at 10.1186/s12884-021-04276-5.

## Background

Expanding maternal healthcare has been a pillar of global efforts to improve pregnancy outcomes and achieve the Sustainable Development Goal of lowering the maternal mortality ratio to less than 70 per 100,000 live births. Antenatal care and skilled childbirth services comprise a suite of maternal healthcare interventions—from health promotion; to disease prevention, screening and diagnosis; to childbirth in the care of skilled birth attendants—all of which save lives and support people during critical times [1]. These interventions are provided across the course of pregnancy, and they require early attendance during pregnancy and frequent follow-up with skilled birth attendants who have training in evidence and human-rights based, quality, dignified care. These skilled birth attendants can facilitate the physiological processes of labor and delivery, and appropriately manage or refer people who experience complications [2]. The best pregnancy and childbirth outcomes are expected to occur when people have access to appropriate care throughout the entire course of pregnancy.

Geographic distance can be a major barrier to achieving care throughout the course of pregnancy. Distance can impact access to care in several different ways. Foremost, people who are in remote areas with limited access to antenatal care may never make it to healthcare facilities. A systematic review of west African countries found that travel distance was a key predictor of the use of maternal and reproductive healthcare services [3], and a prospective cohort study in rural Rwanda found that distance from childbirth services predicts the use of skilled birth attendants but does not affect the number of visits women make to antenatal care facilities [4]. The majority of evidence comes from cross-sectional studies in Cameroon, Rwanda, Ethiopia, Burkina Faso, Ghana, and China which indicate that distance is a major determinant of the use of maternal healthcare services [5–9]. There has been some discordance in these findings. Notably, a spatial analysis from Kenya found that distance did not always correlate with use of antenatal care; highlighting potential contextual and geographic heterogeneity in how distance influences uptake of maternal healthcare [10].

When the travel distance is far or travel is difficult, it can act as a deterrent to seeking reproductive or antenatal care. Whereas some people may never present at a clinic, others will present only late in the pregnancy or after there is a severe medical condition [11, 12]. Distance can also influence the frequency of visits among people who do present at reproductive or antenatal health facilities. Finally, people who must travel far distances to receive care may be more prone toward ceasing regular visits, with their final pregnancy outcome never being documented [4, 13]. Even a 1 km increase in travel distance has been shown to reduce use of skilled birth attendants [14].

Evidence-based interventions have been tested in randomized-controlled trials and have been shown to increase antenatal care access in rural low and lower-middle-income countries [15]. From establishing supply chains for rural villages to acquire tetanus vaccines to improvements in the health worker education system, these interventions have been endorsed by the World Health Organization for their potential to improve maternal health. However, low and lower-middle income countries vary substantially in local contextual factors as well as the factors that determine access to care. As a result, it is difficult to determine which of these evidence-based interventions to apply without further study.

**Fig. 1:**
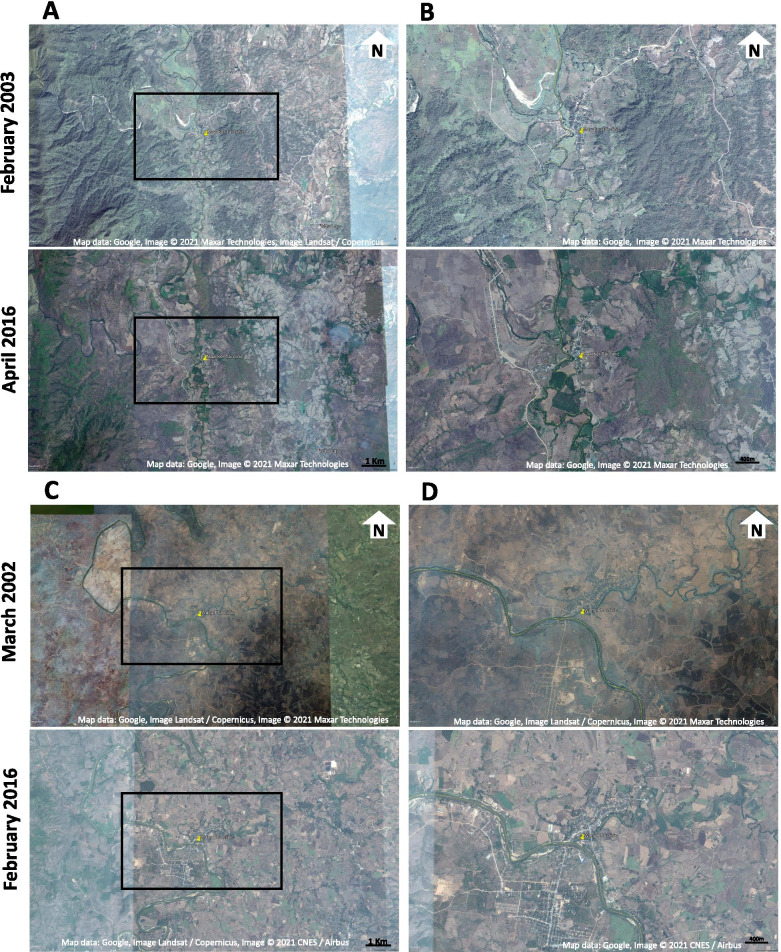
Satellite images showing environmental changes over time for two of the antenatal clinics: Mawker Tai (top panel: **A** and **B**) and Wang Pha (bottom panel: **C** and **D**) clinics. The first columns (**A** for Mawker Tai and **C** for Wang Pha) show the larger geographic area whereas the second columns (**B** and **D**) show greater detail in the immediate clinic area (zoomed areas are indicated with the black boxes in **A** and **C**). The top rows show historical images, and the bottom rows show more recent images. Deforestation is apparent, especially in the Mawker Tai images and on both the Thailand and Myanmar side of the international border. Infrastructure and development have also increased at both clinics, with increased year-round roads, housing, and increased agricultural fields. Images come from Google Earth

The Thailand-Myanmar border region is a complex landscape with displaced populations, migrants, multiple ethnic groups speaking different languages, and many rural, remote communities with general poor access to health services for the many who don’t live in major towns or cities. In eastern Myanmar, where there has been civil conflict now for over half a century, access to health facilities is extremely limited. Until recently, access to many health services was completely lacking and was only accessible to those who travelled by foot to the international border and successfully crossed into Thailand. As conflict has been relatively heterogeneous, stable subregions in eastern Myanmar have begun to build roads (Fig. 1) and clinics but areas that are still politically unstable or with active conflict remain without road systems or modern healthcare facilities. This year’s *coup d’etat* has resulted in widespread breakdown of government healthcare services. On the Thailand side of the border, many have trouble accessing the national healthcare system due to language differences, cost, and a lack of official immigration status. Although the roads are well maintained and local nonprofits provide subsidized transportation, people face some danger of being arrested or fined at road check-points on their way to receive care [16]. On both sides of the Thailand-Myanmar border, many people lack adequate access to reproductive or antenatal services and research on the impact of this lack of access has been hindered by security concerns in eastern Myanmar.

Although data for this conflict-affected population has been difficult to collect, what has been gathered indicates a high burden of poor pregnancy outcomes, mostly driven by conditions that can be reduced or prevented through routine antenatal care and childbirth under the care of skilled birth attendants who possess the required competencies defined by the WHO [2, 17]. Maternal health has also been complicated by endemic malaria in this region, which is a major cause of maternal death, maternal anemia, and adverse pregnancy outcomes [18].

The Shoklo Malaria Research Unit (SMRU) operates a system of antenatal clinics (ANCs) and skilled birth facilities on the Thailand-Myanmar international border that provide free care to border populations from Myanmar and Thailand. We noted high rates of loss to follow-up and high proportions of people first presenting at the clinic in their second or third trimester [17]. Many of the pregnant people reported walking long distances or travelling through difficult conditions, and we therefore hypothesized that geographic distance was influencing access to antenatal services. We also noted that distribution of malaria had changed over time, with a general decrease in *Plasmodium falciparum* infections among patients over the last several decades. Here, we examine associations between distance to clinic and patient’s pregnancy outcomes. We use detailed individual-level clinic records and a newly created geographic information system for Kayin State of Eastern Myanmar to conduct an empirical analysis. Our work will help identify barriers to maternal healthcare and guide implementation of evidence-based interventions. Moreover, our findings can be applied beyond the Thailand-Myanmar border to other rural, remote, and post-conflict settings where comprehensive studies of maternal healthcare access are difficult to conduct.

## Methods

**Fig. 2:**
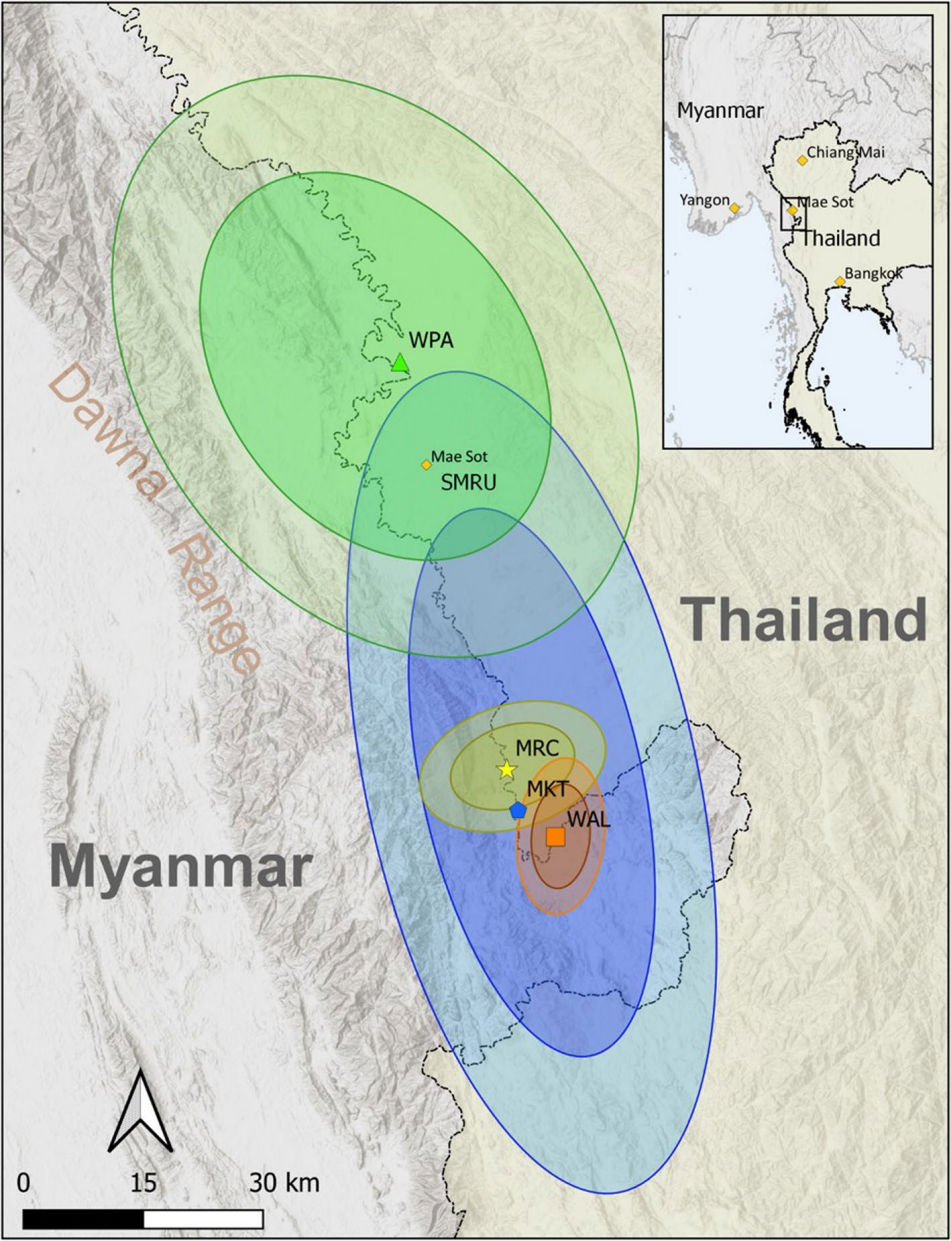
Map of the catchment areas for the four antenatal clinics (ANCs) from 2007 to 2015. Each of the four clinics is indicated by a different color scheme. The ellipses are standard deviational ellipses, with 2 and 3 standard deviations, explained in detail in the supplementary materials. The darker circle represents roughly 98% of people’s home villages for that specific clinic and the lighter circle representing 99.9% of people’s home villages. WPA (green) and MKT (blue) provided both antenatal care and skilled birth attendants, and MRC (yellow) and WAL (orange) provided antenatal care. WPA and MKT had the largest catchment areas whereas MLC and WAL served a more local population. Maps indicating changes in catchment area over time are presented in Supplementary Fig. 2. Maps and layers were created by DMP using QGIS software

SMRU has been based on the Thailand-Myanmar border for over 30 years. In response to an estimated maternal mortality in refugees of 1000 per 100,000 live births in 1985-86, SMRU established a system of weekly ANCs to offer early detection and treatment of *Plasmodium falciparum* malaria in pregnancy [19]. As the population of migrants grew in the 1990’s and 2000’s, SMRU opened four facilities on the international border formed by the Moei River. The facilities provided both antenatal care and childbirth services to local populations who had settled outside of the region’s refugee and internally displaced person camps (Fig. 2). The antenatal care and childbirth services are provided by skilled birth attendants, who are comprised of local staff trained in line with the WHO guidelines to provide evidence and human rights based, quality, dignified care, manage the physiological processes of labor and delivery, and facilitate timely management and referral of complications [2]. These skilled birth attendants conduct health promotion, screening, diagnosis, and administer treatments using evidence-based protocols with licensed physicians available for 24-h medical back up via telephone [20].

These four antenatal clinics on the international border served populations living in both Myanmar and Thailand. Mawker Thai (MKT) began providing ANC and childbirth services in 1998, Walley (WAL) and Mu Ru Chai (MRC) began providing ANC care in 2001, and Wang Pha (WPA) began providing ANC and childbirth services in 2004. WAL closed operations in Jul 2010 and MRC in Dec 2012 and services were amalgamated at MKT. All services were free of charge [21] and attendance at ANCs was voluntary. Trained local sonographers determined gestational age using ultrasound offered at the first antenatal visit [22]. Ultrasound becomes increasingly imprecise at estimating gestational age in those presenting after 24 weeks [1], and so clinical staff derived gestational age from the Dubowitz assessment of gestation at birth, last menstrual period, or symphysis fundal height in people presenting after 24 weeks [23].

The maternal health facilities kept antenatal medical records for each pregnancy from 2007 to 2015 which were de-identified and include general demographic information (patient age, gravidity, parity, home village name, and time lived at home village), antenatal care attendance information (estimated gestational age at initial presentation, miscarriages), pregnancy complication information (malaria infection with *P. vivax*, *P. falciparum*, or both, multiple pregnancy, very young age), and presence of skilled birth attendants at childbirth (loss to follow-up and normal singleton delivery) [24].

Loss to follow-up was defined as a person who enrolled at ANC but then stopped attending and did not return for childbirth. Most people travel on foot to ANC appointments and a minority hire motorbikes or long-tractors for transportation. SMRU partially subsidizes transportation by car to prenatal visits for those who live on the Thai side of the border to MKT and WPA. All four facilities are built on the Thailand bank of the Moei River. Depending on seasonal variations in rainwater people may use temporary bridges or boats to cross the river.

In 2014, SMRU worked to create and update a geographic information system (GIS) database for Kayin state, Myanmar and Tak province, Thailand. In Kayin State, SMRU collaborated with several local community based organizations and travelled to remote villages by car, boat, and foot to obtain coordinates, which was the first systematic geographic study in the area since before World War II [25]. In Tak province, the Tak Malaria Initiative [26] had previously gathered village coordinates (latitude and longitude), and SMRU performed an updated geographic survey in 2014. We used the Kayin and Tak GISs to link each unique pregnancy with the geographic coordinates of their home village.

### Linking geospatial data to de-identified patient data

We used geocoding to convert place names written in patient’s medical records into map coordinates. The geocoders were blinded to all information in the patient record except village names, which they matched with coordinate data in the Kayin and Tak GISs. For the portion of villages not listed in GISs (*n* = 105/1152), ANC clinic administrators with more than 20 years of experience pinpointed villages using Google Earth software. Patient addresses weren’t consistently recorded at ANC clinics until late 2006 and we therefore limited this analysis to 2007 through 2015. We excluded all records that did not include a village name. We also excluded villages that were greater than 35 km from the ANC, given that our accuracy in correctly identifying villages may be better closer to the clinics (a histogram of the distribution of distances is provided in Supplemental Fig. 1).

### Univariate analyses

We estimated the catchment areas for patients attending the respective clinics using standard deviational ellipses (SDE) and calculated the catchment area by converting the SDEs into square kilometers (km2), which we describe in greater detail in Supplemental Text 1 and visualized in Fig. 2 (and Supplemental Fig. 2). Briefly, we generated ellipses for each clinic using the home villages of patients who attended that clinic. The SDE measures two-dimensional spread along an X- and Y-axis from the geometric mean center of a set of points (in this case, home village locations for patients). The Y-axis is rotated until the sum of the squares of the distances between points and axes are minimized. The resulting ellipses provide a visual representation of 63, 98, or 99% of all home villages for 1, 2, or 3 standard deviations, respectively.

In order to investigate potential differences in travel distance we use the straight-line (Euclidian) distance between each patient’s village and the facility where they received care. We calculate distance as the distance between GPS coordinates for the patient’s home village and the clinic they visited, which admittedly does not take into account the ruggedness of terrain or actual road distance. We then calculate univariate and bivariate descriptive statistics for travel distances (minimum distance, maximum distance, first quartile (Q1), third quartile (Q3), and median distance) based on year of childbirth, parity, age, malaria infection status, and pregnancy outcome (i.e., singleton delivery, twins, lost to follow-up, or miscarriage). In our study, we define miscarriage as birth before 28 weeks gestational age. We based this decision on the WHO definition of stillbirth and past studies of the newborn population on the Thailand-Myanmar border [27].

### Negative binomial regression

We use negative binomial regression to formally analyze variables associated with distance to a clinic, and selected distance between home village and clinic (in kilometers) as the outcome variable. Variables in the model included: pregnancy outcome (normal singleton birth, twins, miscarriage, or lost to follow up), the trimester in which the person first presented at the clinic (first, second, or third), country of home village (Thailand or Myanmar), whether or not the person had a *P. falciparum* infection during pregnancy, whether or not the person had a *P. vivax* infection during pregnancy, antenatal clinic where patient received care (MKT clinic, MLC clinic, WAL clinic, WPA clinic) age group (13-14, 15-19, 20-24, 25-29, 30-24, 35-39, and 40+), year of childbirth, parity (0, 1, 2-3, 4-5, 6-9, 10+), and the duration of time the person had lived in their home village (< 1 year, 1-3 years, 4-9 years, 10 or more years). A more detailed description of all the variables we included in the negative binomial regression can be found in Supplemental Table 1.

The output from the negative binomial regression model is a distance ratio (DR), which can be interpreted as a ratio comparing the distance (in km) travelled of one group to a comparison group. For instance, a DR of 1.4 for patients lost to follow-up, in comparison to those with a normal singleton childbirth, indicates that patients lost to follow-up travelled 40% farther than those with normal singleton childbirths after controlling for the other variables in the model. We will henceforth refer to the distance ratio as DR, and we report it alongside a 95% confidence interval (CI).

To check for model sensitivity to potential errors in geocoding, we stratified our negative binomial regression model by distance from the clinic (Supplemental Table 2). We also assess for potential changes in associations between variables over time by stratifying the model by time period (Supplemental Table 3). To assess for differences in our results between people living in Myanmar and Thailand, we also stratify the model by nation of origin (Myanmar or Thailand) (Supplemental Table 4). We also include a variable for the country of home village in the main model to account for important differences between those living in Myanmar and Thailand, including that some people on the Thailand side receive subsidies for transportation to clinic.

### Statistical software

We created maps using QGIS version 3.4.9. We use the Python programming language (version 3.6) to merge geocoded home villages to the patient records, and R statistical software version 3.3.2 for all statistical analyses.

## Results

### Summary statistics and univariate analyses

We identified 17,522 unique pregnancy records from SMRU’s four ANC facilities for the study period of 2007-2015. Thirty percent of the pregnancies (5364/17,522) were lost to follow up and 59% (10,368/17,522) first presented after the first trimester. Those with a singleton or twin birth outcome totaled 10,425 and 1179 had miscarriages. We successfully linked 97.9% of these records (17,162/17,522) to GPS coordinates in the GIS databases. After dropping records with patients who lived ≥35 km away from the clinic and any record with missing information we had 11,624 unique, fully complete records. The most common missing information was for the number of years lived in the home village, which was not regularly recorded until 2009.

Figure 2 shows the estimated catchment areas for each of the four ANC facilities, and Supplementary Fig. 2 shows changes in clinic catchment area over time. The estimated catchment areas from the standard deviational ellipses show that 99% of all patients at WPA lived within an area of 3416 km^2^; for WAL it was an area of 161 km^2^; for MLC it was an area of 276 km^2^; and for MKT it was an area of 3256 km^2^. At the beginning of the study period the catchment area was relatively small, with most people traveling from nearby (Supplementary Table 5 and Supplementary Fig. 2). Over time, the median travel distance increased. We noted specific increases around June 2010, when SMRU transferred care of WAL patients to MKT, and in December 2012, when SMRU transferred care of MRC patients to MKT (Supplementary Table 5).

Notably, travel distance was associated with maternal health service use. People lost to follow-up travelled 33% farther compared to people who followed-up for normal singleton deliveries in the presence of a skilled birth attendant (median 8.0 km versus 6.0 km) (Table 1). Those who first received antenatal care in the third trimester travelled a longer median distance compared to those who began antenatal care in the first and second trimesters (median 8.0 km versus 6.0 km﻿ or 7.0 km, respectively; Table 1). For people with *P. falciparum* malaria in pregnancy, they travelled similar distances compared to those without malaria infections (median 6.0 km versus 6.0 km for those who never had malaria in their pregnancy) (Table 1).

**Table 1:** Distance from home village (in km) to health facility by different variables of interest (year of childbirth; trimester of first attendance; pregnancy outcome; age; and malaria status)

	Minimum	1st quartile	Median	3rd quartile	Maximum	Number of people
**Pregnancy Outcome**						
Singleton delivery	0.0	1.0	6.0	10.0	34.0	7268
Lost to follow-up	0.0	5.0	8.0	16.0	34.0	3385
Miscarriage	0.0	1.0	6.0	10.0	34.0	879
Twins delivery	0.0	2.0	7.0	12.3	31.0	92
**Trimester of first attendance**						
1st	0.0	1.0	6.0	9.0	34.0	4328
2nd	0.0	2.0	7.0	12.0	34.0	4935
3rd	0.0	4.0	8.0	16.0	34.0	2361
**Malaria status**						
No malaria	0.0	0.9	6.0	9.4	33.8	10,713
*P. falciparum*	0.0	1.1	6.0	8.9	33.8	247
*P. vivax*	0.0	0.9	4.7	7.4	31.0	718
Mixed^a^	0.0	0.9	5.8	7.6	30.6	54

^a^Mixed infections are counted in both *P. falciparum* and *P. vivax*, therefore the sum for malaria status is greater than total number of patients in the data

### Results from the negative binomial regression model

Pregnant people lost to follow-up travelled 50% farther than people who followed-up for normal singleton deliveries in the presence of a skilled birth attendant (DR: 1.5; CI: 1.4 – 1.5) (Table 2), after controlling for all other variables in the model. Likewise, people who first presented for care in their third trimester came from 50% farther than those who presented in their first trimester (DR: 1.5; CI: 1.4 – 1.5) (Table 2), and those with homes in Thailand travelled 60% farther than those with homes in Myanmar to receive care at ANCs (DR: 1.6; CI: 1.6- 1.7) (Table 2).

**Table 2:** Results from a negative binomial regression for predictors of distance to the health facility. The results are given as a ratio of the distances traveled (i.e., the distance ratio (DR)) between a variable and its comparison

Covariate	Count	DR (95% CI)
*Pregnancy Outcome*		
Normal singleton delivery	7268	
Lost to follow-up	3385	1.5 (1.4–1.5)
Miscarriage	879	1.2 (1.1–1.3)
Twins delivery	92	1.2 (1.0–1.5)
*Trimester at first visit to ANC*		
1st trimester presentation	4328	
2nd trimester presentation	4935	1.2 (1.2–1.3)
3rd trimester presentation	2361	1.5 (1.4–1.5)
*Country of home village*		
Home village in Myanmar	6523	
Home village in Thailand	5101	1.6 (1.6–1.7)
*Malaria infections during pregnancy*		
No *P. falciparum* infection	11,377	
*P. falciparum* infection	247	1.6 (1.4–1.8)
No *P. vivax*	10,906	
*P. vivax* infection	718	1.2 (1.2–1.4)
*Health facility where patient received care*		
MKT clinic	3930	
MLC clinic	495	0.5 (0.5–0.6)
WAL clinic	141	0.4 (0.3–0.5)
WPA clinic	7058	1.0(1.0–1.2)
*Patient age at initial ANC visit*		
13–14	9	
15–19	1805	0.8 (0.4–1.4)
20–24	3419	0.8 (0.4–1.4)
25–29	2629	0.8 (0.4–1.4)
30–34	1901	0.8 (0.4–1.4)
35–39	1285	0.7 (0.4–1.2)
40 +	576	0.7 (0.4–1.3)
*Patient birth date*		
Year of childbirth		1.1 (1.0–1.1)
*Patient parity*		
Parity: 0	4172	
Parity: 1	2634	1.0 (0.9–1.0)
Parity: 2–3	3088	1.0 (1.0–1.1)
Parity: 4–5	1257	1.1 (1.0–1.2)
Parity: 6–9	450	1.1 (1.0–1.3)
Parity: 10+	23	1.1 (0.8–1.7)
*Patient’s years lived in their home village at time of initial ANC visit*		
Lived in home village for less than 1 year	4654	
Lived in home village for 1 through 3 years	2797	1.1 (1.1–1.2)
Lived in home village for 4 through 9 years	2524	1.0 (0.9–1.0)
Lived in home village for 10 or more years	1649	1.7 (1.6–1.7)

^a^Patients with incomplete records were dropped from this analysis

Specific groups requiring more specialized care in pregnancy also travelled longer distances. People experiencing miscarriage came from 20% farther away than those who had normal singleton deliveries (DR: 1.2; CI: 1.1-1.3). People with *P. falciparum* malaria came from 60% farther away (DR: 1.6; CI: 1.4 – 1.8) than people without *P. falciparum* (Table 2), and those with *P. vivax* malaria travelled 20% farther than those without *P. vivax* (DR: 1.2; CI 1.2-1.4).

In our sensitivity analysis, we consistently found the same associations mentioned above after stratifying our model by travel distance (Supplemental Table 2), time (Supplemental Table 3), and nation of origin (Supplemental Table 4). When we stratified by nation of origin, however, we found that people living in Thailand had no association between travel distance and malaria infection status while people living in Myanmar consistently travelled longer distances if they had *P. falciparum* malaria or *P. vivax* malaria in pregnancy (Supplemental Table 4).

## Discussion

These results add to a growing body of literature, mainly gathered in low-income and resource-limited settings, that highlight how travel distance limits access to maternal healthcare in several ways. From longer travel distances amongst pregnant people lost to follow-up [11, 14, 28] to an absence of early antenatal care due to late presentation [29] to longer travel distances amongst pregnant people with malaria [6, 30], distance to health services is associated with myriad disruptions in providing antenatal healthcare.

This analysis presents the first empirical evidence that travel distance contributes to the high proportion of patients lost to follow-up after enrolling in antenatal care on the Thailand-Myanmar border. Given the paucity of any other antenatal care or childbirth services for migrants in the rural Thailand-Myanmar border region, it is likely that most of those lost to follow-up went without antenatal care and gave birth at home without a skilled attendant. Maternal and neonatal mortality are known to increase when people do not receive antenatal care [1] and/or deliver without skilled birth attendants. Although birth outcomes amongst people lost to follow-up in our study were not available, they are at a higher risk of pregnancy-related morbidity and mortality compared to those who attend ANCs and delivery units for care [31].

Systematic reviews have found moderate quality evidence that health interventions may increase the number of ANC visits and deliveries in health facilities [15]. Employing one of those interventions—from re-organization of health services to health worker education to mass media campaigns—may help to lower the number of people lost to follow-up and thereby improve birth outcomes in the Thailand-Myanmar border region.

This study also identified an association between longer distance travelled and late presentation for antenatal care, indicating limited access to mortality-reducing interventions amongst those living far from health facilities. Increased outreach services to enroll this population in ANC care during the first trimester will broaden access to iron supplements to treat anemia, and HIV antiretrovirals to prevent maternal-newborn transmission: these are just a few of the interventions that have moderate evidence of reducing perinatal mortality and preventing low birthweight according to a recent systematic review [15]. That 59% of the study population (9761/16,548; from Table 2) did not present until after the first trimester of pregnancy also suggests the need for clinical guidelines for antenatal care for those who miss their first trimester antenatal appointments.

This study also identified an association between longer distance travelled and people experiencing miscarriage. Miscarriage can occur in as many as 10% of recognized pregnancies [2], but treatment may require frequent follow-up for serial laboratory testing to rule out life-threatening complications like ectopic pregnancies. Future interventions should offer specialized care coordination and travel subsidies to help prevent loss to follow-up and to increase access to the specialized services necessary for the management of miscarriage.

The finding that people with malaria travel longer distances to access care may be related to the high rates of loss to follow-up in people with malaria, noted by Moore et al. [32]. Stratified analysis suggests that this finding is restricted to those living on the Myanmar side of the border (Supplementary Table 4). These differences by country of residence may be accounted for by both the decreased burden of *P. falciparum* malaria in Thailand after decades of concerted public health efforts as well as the increased access to care in Thailand provided by subsidized transportation to ANC. Geographically, the highest risk of malaria transmission in Thailand exists close to the border. Thai-based people infected with malaria are therefore likely to live close to the clinics, which are located on the border. Historically, Thailand has provided free access to diagnosis and treatment of malaria regardless of nationality, and people from Myanmar have crossed the international border to receive care. This may have decreased the burden of *P. falciparum* malaria in Myanmar communities very near the border and offers an explanation for why the association between longer travel distance and malaria infection disappears amongst those living within 5 km of the antenatal clinics (Supplemental Table 2). Since 2014 SMRU has expanded access to malaria diagnosis and treatment in eastern Myanmar as well [25], though our model does not show any change in travel distance amongst those with malaria after it was implemented (Supplemental Table 3).

Treatment of malaria in pregnancy has been a major priority for preventing poor pregnancy outcomes worldwide, but the Thailand-Myanmar border region faces unique challenges due to high rates of *P. falciparum* multi drug resistance [33] and the lack of a safe, radical cure that eliminates dormant *P. vivax* from the liver in pregnancy [34]. Recent initiatives have made progress by bringing curative *P. falciparum* treatments to rural villages [25], but *P. vivax* now accounts for the majority of malaria infections in this region and provision of radical curative treatments is hampered by the high rate of G6PD deficiency in this population [34]. Public health campaigns to address the burden of *P. vivax* must focus on population-wide *P. vivax* screening and treatment of all non-pregnant residents, which is arguably the most effective tool to decrease the prevalence of *P. vivax* malaria in pregnancy in this region [17].

Although these findings highlight poor access to care amongst those living far from ANC, they also demonstrate the remarkable resilience of people living in the Thailand-Myanmar border region. Facing high fevers from malaria and other complications of pregnancy, people travel long distances by any means available (foot, boat, tractor, motorboat, or truck being most common) and overcome significant geographic barriers to receive healthcare.

These analyses and data are subject to several limitations. First, there are no officially numbered houses or named streets in the study area, and the addresses we use in this analysis correspond to village names. The sensitivity analysis (Supplemental Table 2) provides some confidence in the validity of the geocoding approach used to identify home villages. However, our data prevents us from conducting a more micro-scale analysis at the sub-village level due to the lack of official addresses. The study population also includes people whose home location can change seasonally based on employment opportunities, and the address we use only reflects where a person lived at the time of their initial ANC consultation. We do, however, attempt to control for movement by including the duration of time lived at the current address as a variable in our regression analysis.

Furthermore, the pregnant people who never sought healthcare services are not represented in our data. This could introduce a selection bias for a healthier patient population. Past studies in this region have documented a significantly higher maternal mortality ratio amongst those living in rural villages far from maternal health services (721 deaths per 100,000 live births) [35], compared to people that receive antenatal care and childbirth services (250 deaths per 100,000 live births) [17]. Our study is therefore at risk of excluding people at the highest risk of poor pregnancy outcomes.

Finally, we use straight-line distance as a proxy for geographic access, which does not account for geographic factors like mountain ranges, rivers, and seasonal variations in rains that can wash out dirt roads and make travel arduous. Straight-line distance and travel time have been found to closely correlate in other limited-resource settings, but no studies have examined whether that relationship holds in the Thailand-Myanmar border region. The straight-line distance represents the easiest possible travel pathway, with reality being that travel is much more difficult for the people in this study. The straight-line distance may have a slightly different relationship to the true distance traveled in Myanmar (with very limited infrastructure) vs. Thailand (where roads are quite good).

Though there are limitations to this analysis, there are many strengths as well. We collected data from a longitudinal cohort of people followed from pregnancy through childbirth and maintained the data despite floods and armed conflicts. Although most studies on distance and access to maternal healthcare are survey-based or cross-sectional, this prospectively followed cohort provides richer detail on healthcare utilization throughout pregnancy and across time. Further, this analysis harnesses recently created geographic information systems to understand the maternal and child health landscape in the Thailand-Myanmar border region for the first time.

## Conclusions

We recommend two further lines of research in this region. Quality of care may impact travel distances and has recently been shown to have a pronounced effect on decisions of where to seek care in other resource-limited settings [36]. A more nuanced analysis of distance including quality of care measures would build a more complete picture of the factors underlying the decision to seek care. Second, this study identified that people in Thailand travelled 63% farther than people in Myanmar for antenatal services. Future studies can clarify whether this difference is related to the subsidized transport provided to Thailand residents by SMRU, the better roads and transportation infrastructure in Thailand, or other factors like availability of alternative healthcare services.

Given continued poor access to antenatal services for many remote communities in eastern Myanmar and recent escalations in armed conflict, there will continue to be a substantial population in need of antenatal services in this region for the foreseeable future. As of April of 2021 there are an estimated 24,000 newly displaced persons in the catchment areas of SMRU’s antenatal clinics [37]. The findings from our study speak to the need to implement evidence-based interventions to increase access to antenatal and childbirth services. Moreover, continued support for the Thailand-Myanmar border region’s public health infrastructure will help monitor health outcomes and enable faster interventions that will help improve maternal health outcomes in the Thailand-Myanmar border region.

## Supplementary Information

**Additional file 1: Supplemental Text 1.** Estimating clinic catchment areas (standard deviational ellipses). **Supplementary Figure 1.** Histogram of distances to antenatal (ANC) clinics. **Supplementary Figure 2.** Maps indicating catchment areas for the clinics over time. **Supplementary Table 1.** Table of variables. **Supplementary Table 2.** Negative binomial regression stratified by distance (sensitivity analysis). **Supplementary Table 3.** Negative binomial regression stratified by time. **Supplementary Table 4.** Negative binomial regression stratified by nation of origin (Thailand or Myanmar). **Supplementary Table 5.** Summary statistics for distance to clinic by year and clinic. **Supplementary Table 6.** Summary statistics for number of consultations (NOC) by distance.

## Data Availability

Access to the data used in this analysis can be requested through the Mahidol-Oxford Tropical Medicine Research Unit data access policy. Both the policy and application form are available at: http://www.tropmedres.ac/data-sharing.

## Abbreviations

ANC: Antenatal clinic
GIS: Geographic information system
MKT: Mawker Thai clinic
MRC: MuRu Chai clinic
SMRU: Shoklo Malaria Research Unit
WAL: Walley clinic
WPA: Wang Pha clinic

## References

[1] World Health Organization (2016). WHO recommendations on antenatal care for a positive pregnancy experience.

[2] Organization WH (2018). Defining competent maternal and newborn health professionals: background document to the 2018 joint statement by WHO, UNFPA, UNICEF, ICM, ICN, FIGO and IPA: definition of skilled health personnel providing care during childbirth.

[3] Ayanore MA, Pavlova M, Groot W (2016). Unmet reproductive health needs among women in some west African countries: a systematic review of outcome measures and determinants. Reprod Health.

[4] Nathan LM, Shi Q, Plewniak K (2015). Decentralizing maternity services to increase skilled attendance at birth and antenatal care utilization in rural Rwanda: a prospective cohort study. Matern Child Health J.

[5] Nisingizwe MP, Tuyisenge G, Hategeka C, Karim ME (2020). Are perceived barriers to accessing health care associated with inadequate antenatal care visits among women of reproductive age in Rwanda?. BMC Pregnancy Childbirth.

[6] Ouedraogo M, Kurji J, Abebe L, et al. Utilization of key preventive measures for pregnancy complications and malaria among women in Jimma zone, Ethiopia. BMC Public Health. 2019;19. 10.1186/s12889-019-7727-8. 10.1186/s12889-019-7727-8 PMC6827171 31684923

[7] You H, Yu T, Gu H, et al. Factors associated with prescribed antenatal care utilization: a cross-sectional study in eastern rural China. Inquiry. 2019. 10.1177/0046958019865435. 10.1177/0046958019865435 PMC6681245 31370723

[8] Mwase T, Brenner S, Mazalale J (2018). Inequities and their determinants in coverage of maternal health services in Burkina Faso. Int J Equity Health.

[9] Asundep NN, Carson AP, Turpin CA (2013). Determinants of access to antenatal care and birth outcomes in Kumasi, Ghana. J Epidemiol Glob Health.

[10] Prudhomme O’Meara W, Platt A, Naanyu V, Cole D, Ndege S (2013). Spatial autocorrelation in uptake of antenatal care and relationship to individual, household and village-level factors: results from a community-based survey of pregnant women in six districts in western Kenya. Int J Health Geogr.

[11] Ganatra BR, Coyaji KJ, Rao VN (1998). Too far, too little, too late: a community-based case-control study of maternal mortality in rural west Maharashtra, India. Bull World Health Organ.

[12] Thaddeus S, Maine D (1994). Too far to walk: maternal mortality in context. Soc Sci Med.

[13] Rosário EVN, Gomes MC, Brito M, Costa D (2019). Determinants of maternal health care and birth outcome in the Dande Health and Demographic Surveillance System area, Angola. PLoS One.

[14] Dotse-Gborgbortsi W, Dwomoh D, Alegana V, Hill A, Tatem AJ, Wright J (2020). The influence of distance and quality on utilisation of birthing services at health facilities in eastern region, Ghana. BMJ Glob Health.

[15] Mbuagbaw L, Medley N, Darzi AJ, Richardson M, Habiba Garga K, Ongolo-Zogo P. Health system and community level interventions for improving antenatal care coverage and health outcomes. Cochrane Database Syst Rev. 2015:1–157. 10.1002/14651858.CD010994.pub2. 10.1002/14651858.CD010994.pub2 PMC4676908 26621223

[16] Tschirhart N, Jiraporncharoen W, Angkurawaranon C (2020). Choosing where to give birth: factors influencing migrant women’s decision making in two regions of Thailand. PLoS One.

[17] McGready R, Boel M, Rijken MJ (2012). Effect of early detection and treatment on malaria related maternal mortality on the north-western border of Thailand 1986–2010. PLoS One.

[18] Malaria in pregnancy. Maternal Health Task Force. 2015. https://www.mhtf.org/topics/malaria-in-pregnancy/. Accessed 2 May 2021.

[19] Nosten F, ter Kuile F, Maelankirri L, Decludt B, White NJ (1991). Malaria during pregnancy in an area of unstable endemicity. Trans R Soc Trop Med Hyg.

[20] White AL, Min TH, Gross MM (2016). Accelerated training of skilled birth attendants in a marginalized population on the Thai-Myanmar border: a multiple methods program evaluation. PLoS One.

[21] Carrara VI, Lwin KM, Phyo AP, et al. Malaria burden and artemisinin resistance in the mobile and migrant population on the Thai–Myanmar border, 1999–2011: an observational study. PLoS Med. 2013;10. 10.1371/journal.pmed.1001398. 10.1371/journal.pmed.1001398 PMC3589269 23472056

[22] Rijken MJ, Mulder EJH, Papageorghiou AT (2012). Quality of ultrasound biometry obtained by local health workers in a refugee camp on the Thai–Burmese border. Ultrasound Obstet Gynecol.

[23] Moore KA, Simpson JA, Thomas KH (2015). Estimating gestational age in late presenters to antenatal care in a resource-limited setting on the Thai-Myanmar border. PLoS One.

[24] Parker AL, Parker DM, Zan BN (2018). Trends and birth outcomes in adolescent refugees and migrants on the Thailand-Myanmar border, 1986-2016: an observational study. Wellcome Open Res.

[25] Parker DM, Landier J, Thu AM (2017). Scale up of a plasmodium falciparum elimination program and surveillance system in Kayin State, Myanmar. Wellcome Open Res.

[26] Carrara VI, Sirilak S, Thonglairuam J, et al. Deployment of early diagnosis and mefloquine- artesunate treatment of falciparum malaria in Thailand: the Tak Malaria Initiative. PLoS Med. 2006:3. 10.1371/journal.pmed.0030183. 10.1371/journal.pmed.0030183 PMC1470664 16719547

[27] McGready R, Paw MK, Wiladphaingern J (2016). The overlap between miscarriage and extreme preterm birth in a limited-resource setting on the Thailand-Myanmar border: a population cohort study. Wellcome Open Res.

[28] Nesbitt RC, Lohela TJ, Soremekun S (2016). The influence of distance and quality of care on place of delivery in rural Ghana. Sci Rep.

[29] Simkhada B, van Teijlingen ER, Porter M, Simkhada P (2008). Factors affecting the utilization of antenatal care in developing countries: systematic review of the literature. J Adv Nurs.

[30] Gupta S, Yamada G, Mpembeni R (2014). Factors associated with four or more antenatal care visits and its decline among pregnant women in Tanzania between 1999 and 2010. PLoS One.

[31] Grzybowski S, Stoll K, Kornelsen J (2011). Distance matters: a population based study examining access to maternity services for rural women. BMC Health Serv Res.

[32] Moore KA, Simpson JA, Paw MK (2016). Safety of artemisinins in first trimester of prospectively followed pregnancies: an observational study. Lancet Infect Dis.

[33] Ashley EA, Dhorda M, Fairhurst RM (2014). Spread of artemisinin resistance in plasmodium falciparum malaria. N Engl J Med.

[34] Chu CS, Carrara VI, Parker DM (2020). Declining burden of plasmodium vivax in a population in northwestern Thailand from 1995 to 2016 before comprehensive primaquine prescription for radical cure. Am J Trop Med Hyg.

[35] Loyer AB, Ali M, Loyer D (2014). New politics, an opportunity for maternal health advancement in eastern Myanmar: an integrative review. J Health Popul Nutr.

[36] Mubiri P, Kajjo D, Okuga M (2020). Bypassing or successful referral? A population-based study of reasons why women travel far for childbirth in eastern Uganda. BMC Pregnancy Childbirth.

[37] Burma army continues to kill daily across Burma. Free Burma rangers. 2021. https://www.freeburmarangers.org/2021/04/21/burma-army-continues-kill-daily-across-burma/. Accessed 27 Apr 2021.

